# Changing haemodynamic status of patients referred for transcatheter aortic valve intervention during the COVID-19 pandemic

**DOI:** 10.1007/s12471-023-01795-y

**Published:** 2023-07-27

**Authors:** Joris F. Ooms, Thijmen W. Hokken, Rik Adrichem, Dilay Gunes, Marjo de Ronde-Tillmans, Isabella Kardys, Jeannette Goudzwaard, Francesco Mattace-Raso, Rutger-Jan Nuis, Joost Daemen, Nicolas M. Van Mieghem

**Affiliations:** 1 Department of Interventional Cardiology, Thoraxcenter, Erasmus University Medical Centre, Rotterdam, The Netherlands; 2 Department of Internal Medicine, Section of Geriatrics, Erasmus University Medical Centre, Rotterdam, The Netherlands

**Keywords:** Transcatheter aortic valve implantation, Haemodynamics, COVID-19

## Abstract

**Introduction:**

Delays in the diagnosis and referral of aortic stenosis (AS) during the coronavirus disease 2019 (COVID-19) pandemic may have affected the haemodynamic status of AS patients. We aimed to compare clinical and haemodynamic characteristics of severe AS patients referred for transcatheter aortic valve implantation (TAVI) or balloon aortic valvuloplasty (BAV) before the pandemic versus two subsequent periods.

**Methods:**

This study compared three 1‑year historical cohorts: a pre-COVID-19 group (PCOV), a 1st-year COVID-19 group (COV-Y1) and a 2nd-year COVID-19 group (COV-Y2). The main parameters were baseline New York Heart Association (NYHA) functional class, left ventricular ejection fraction (LVEF) and left ventricular end-diastolic pressure (LVEDP). Demographics, procedural characteristics and 30-day clinical outcomes were assessed. The transition time between heart team decision and TAVI was examined. Pairwise group comparisons were performed (PCOV vs COV-1Y and COV-1Y vs COV-2Y).

**Results:**

A total of 720 patients were included with 266, 249 and 205 patients in the PCOV, COV-Y1 and COV-Y2 groups, respectively. BAV was performed in 28 patients (4%). NYHA class did not differ across the cohorts. Compared to PCOV, LVEF was slightly lower in COV-Y1 (58% (49–60%) vs 57% (45–60%), *p* = 0.03); no difference was observed when comparing COV-Y1 and COV-Y2. LVEDP was higher in COV-Y1 than in PCOV (20 mm Hg (16–26 mm Hg) vs 17 mm Hg (13–24 mm Hg), *p* = 0.01). No difference was found when comparing LVEDP between COV-Y1 and COV-Y2. Thirty-day mortality did not differ between groups. Transition time was reduced in the COVID era. Duration of hospital stay declined over the study period.

**Conclusions:**

Patients undergoing TAVI during the COVID-19 pandemic had more advanced AS illustrated by lower LVEF and higher LVEDP, but there were no differences in clinical outcome. The TAVI pathway became more efficient.

**Supplementary Information:**

The online version of this article (10.1007/s12471-023-01795-y) contains supplementary material, which is available to authorized users.

## Whats new?

- The haemodynamic status of patients with severe aortic stenosis undergoing transcatheter aortic valve intervention (TAVI) was different in the COVID-19 era, with lower left ventricular ejection fraction and higher left ventricular end-diastolic pressure.
- During the COVID-19 pandemic, the need for admission to an intensive care unit post-TAVI became negligible and overall length of hospital stay shortened.
- The transition time from heart team discussion to TAVI procedure was reduced throughout the COVID-19 period, suggesting a more efficient TAVI pathway.

## Introduction

Transcatheter aortic valve implantation (TAVI) has matured into an established treatment strategy for elderly patients with symptomatic severe aortic stenosis (AS) [1]. Symptomatic severe AS has a 25–50% 1‑year mortality rate if left untreated [2]. Furthermore, decompensated AS is associated with incremental mortality risks before, during and after valve replacement therapy [3, 4].

The coronavirus disease 2019 (COVID-19) pandemic has caused unprecedented strain on healthcare systems globally. Re-allocation of resources included postponement of elective, non-emergent procedures such as aortic valve replacement for severe AS. De-escalation of outpatient clinic activities, including fewer transthoracic echocardiography (TTE) studies, and promotion of telehealth may further delay the diagnosis of severe AS and affect the clinical and haemodynamic status of patients with symptomatic severe AS. Equally important, a streamlined TAVI pathway that minimises hospital transition times may ensure maintained care of severe AS patients and precludes interference with COVID-19 logistics. Therefore, our aim was to compare clinical and haemodynamic characteristics of the patients who underwent TAVI or balloon aortic valvuloplasty (BAV) in the year before the COVID outbreak, the 1st year and the 2nd year of the COVID pandemic. Additionally, we looked at patient transition times from hospital referral to hospital discharge, length of hospital stay and 30-day outcomes across the different time cohorts.

**Fig. 1:**
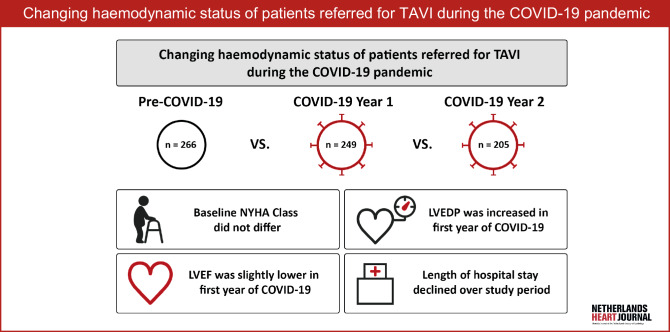
Infographic. A single center observational study comparing clinical and hemodynamic characteristics of three successive one-year historical cohorts of TAVI patients. *LVEDP* left ventricular end diastolic pressure, *LVEF* left ventricular ejection fraction, *NYHA* New York Heart Association functional classification, *TAVI* transcatheter aortic valve implantation

## Methods

The first case of COVID-19 in the Netherlands was confirmed on 27 February 2020 [5]. We collected baseline demographics and clinical, echocardiographic and haemodynamic characteristics of all consecutive patients with severe AS or a degenerated bioprosthesis referred for transcatheter aortic valve treatment (i.e. TAVI or BAV) between 27 February 2019 and 27 February 2022. Three cohorts were determined according to time period: cohort 1 (PCOV), preceding the COVID-19 outbreak, ranged from 27 February 2019 to 26 February 2020; cohort 2 (COV-Y1), the 1st year of COVID-19, ranged from 27 February 2020 to 26 February 2021; and cohort 3 (COV-Y2) ranged from 27 February 2021 to 27 February 2022.

A multidisciplinary heart team that included interventional cardiologists, cardiac imagers, cardiac surgeons and geriatricians reached a consensus for transcatheter aortic valve treatment for all patients. Patient frailty was determined by multi-parametric geriatric assessment (including hand grip, gait speed, mini-mental state examination and instrumental activities of daily living) and verified by the heart team. Procedural planning included multi-slice computed tomography scanning of the aortic valve and arterial tree, TTE and invasive coronary angiography. TAVI procedures were performed via (predominantly) transfemoral or transaxillary arterial access with the patient under local anaesthesia. Filter-based cerebral embolic protection was used whenever anatomically feasible. Procedural details regarding our BAV procedure have been described elsewhere [6]. Invasive haemodynamics included measurement of the left ventricular end-diastolic pressure (LVEDP), maximum and mean transaortic gradient and heart-rate-adjusted aortic regurgitation index (ARi). The heart-rate-adjusted ARi is calculated by dividing the difference between the aortic diastolic blood pressure and LVEDP by the heart rate and multiplying this number by 80 [7].

The main study objectives were to compare among the three cohorts: (1) baseline New York Heart Association (NYHA) functional class, (2) left ventricular ejection fraction (LVEF) as determined by echocardiography and (3) LVEDP. We also collected the patient’s clinical status at hospital admission and defined decompensated AS as an ongoing hospitalisation for heart failure (HF)-related symptoms that precluded discharge prior to TAVI. We used the Valve Academic Research Consortium 3 consensus (VARC-3) definitions for all clinical outcomes at 30-day follow-up that included all-cause mortality, all stroke, major bleeding, major vascular complication, cardiac structural complication and acute kidney injury [8]. Aortic regurgitation (AR) was assessed at discharge TTE. TAVI pathway efficiency was expressed by transition time and overall length of hospital stay. Transition time was set as the time from discussion in the heart team to TAVI procedure day. Hospital stay lasted from TAVI procedure day to hospital discharge. Other study parameters included baseline demographics, HF medication use, TTE and procedural data.

All patients provided written informed consent for the intervention and subsequent data analysis for research purposes. The study complied with the principles of the Declaration of Helsinki and did not fall under the scope of the Medical Research Involving Human Subjects Act as judged by the Institutional Review Board of the Erasmus Medical Centre.

### Statistical analysis

The distribution of continuous variables was tested for normality by performing a Shapiro-Wilk test. Continuous variables were reported as mean with standard deviation or median with 25th and 75t h percentile depending on normality. Categorical variables were reported as numbers and percentages. Pre-specified pairwise comparisons were used to analyse group differences. PCOV was compared with COV-Y1, and COV-Y1 with COV-Y2. Depending on variable type and distribution, independent *t*-tests, Mann-Whitney U tests, chi-square or Fisher’s exact tests were used. A two-sided *p* < 0.05 was considered statistically significant. All statistical analyses were performed with SPSS 25.0 (IBM, Armonk, NY, USA).

## Results

A total of 720 patients were included in this study. Median age was 80 (74–84) years and 54% were male. Overall median Euroscore II was 2.6% (1.6–5.0%), clinical frailty was present in 47%, and 37% of patients were walking aid dependent. A total of 32 (4%) patients had a degenerated aortic bioprosthesis. The PCOV, COV-Y1 and COV-Y2 groups comprised 266 (37%), 249 (35%) and 205 (28%) patients, respectively (Fig. 1). Baseline demographics and echocardiographic data are presented in Tab. 1. Compared to PCOV, patients in the COV-Y1 cohort were more often male and had higher rates of pacemaker/ICD at baseline. Peripheral artery disease (PAD) and clinical frailty were more prevalent in the PCOV group. NYHA functional class and hospitalisations for HF were similar across the study cohorts. Details on NYHA and HF medication are provided in Tables S1 and S2 (Electronic Supplementary Material). Baseline echocardiographic LVEF data were available in 99% of patients. LVEF was significantly lower in COV-Y1 compared to the PCOV group (57% (45–60%) vs 58% (49–60%), *p* = 0.03), while no difference was found between the COV-Y1 and COV-Y2 groups (57% (45–60%) vs 57% (50–60%), *p* = 0.52).

**Table 1:** Baseline characteristics and echocardiographic parameters

	Pre-COVID-19 *n* = 266	COVID-19 year 1 *n* = 249	COVID-19 year 2 *n* = 205	*p*-value (P-1)	*p*-value (P-2)
*Baseline characteristics*					
Age, years	80 (74–85)	79 (74–83)	79 (74–84)	0.07	0.76
Male	137 (51.5)	151 (60.6)	98 (47.8)	0.04	0.01
Hypertension	201 (75.6)	189 (75.9)	156 (76.1)	0.93	0.96
Diabetes mellitus	84 (31.6)	79 (31.7)	44 (21.5)	0.97	0.01
Stroke/TIA	61 (22.9)	49 (19.7)	54 (26.3)	0.37	0.09
Peripheral artery disease	66 (24.8)	43 (17.3)	24 (11.7)	0.04	0.10
COPD	41 (15.4)	30 (12.0)	27 (13.2)	0.27	0.72
Previous myocardial infarction	37 (13.9)	39 (15.7)	31 (15.1)	0.58	0.87
Previous PCI	72 (27.1)	58 (23.3)	52 (25.4)	0.32	0.61
Previous CABG	25 (9.4)	22 (8.8)	21 (10.2)	0.82	0.61
Previous aortic valve surgery	10 (3.8)	7 (2.8)	8 (3.9)	0.55	0.52
Previous TAVI	3 (1.1)	1 (0.4)	3 (1.5)	0.35	0.23
Atrial fibrillation	94 (35.3)	84 (33.7)	63 (30.7)	0.70	0.50
Permanent pacemaker/ICD	27 (10.2)	42 (16.9)	19 (9.3)	0.03	0.02
EURO II score, %	2.5 (1.7–5.2)	2.6 (1.5–5.3)	2.7 (1.6–4.3)	0.91	0.82
Creatinine clearance, ml/min	63 (50–78)	62 (50–77)	64 (53–77)	0.93	0.67
NYHA functional class IV^a^	47 (18.0)	56 (22.6)	42 (20.5)	0.20	0.54
Admitted with decompensated AS at baseline	43 (12.8)	43 (17.3)	31 (15.1)	0.15	0.54
Any HF hospitalisation	90 (33.8)	90 (36.1)	66 (32.2)	0.58	0.38
Clinical frailty^b^	140 (52.6)	108 (43.4)	91 (44.4)	0.04	0.83
Walking aid dependent	94 (35.3)	92 (36.9)	80 (39.0)	0.70	0.65
*Echocardiographic parameters*					
LVEF, %	58 (49–60)	57 (45–60)	57 (50–60)	0.03	0.52
Aortic valve MG, mm Hg	38 (31–47)	39 (30–48)	40 (32–49)	0.94	0.16
Aortic valve area, cm^2^	0.8 (0.7–0.9)	0.8 (0.6–0.9)	0.7 (0.6–0.9)	0.29	0.27
Aortic regurgitation ≥ moderate	40 (15.0)	24 (9.6)	26 (12.7)	0.06	0.30
Mitral regurgitation ≥ moderate	54 (20.3)	43 (17.3)	41 (20.0)	0.38	0.46
Tricuspid regurgitation ≥ moderate	33 (12.4)	41 (16.5)	27 (13.2)	0.19	0.33

Values are numbers (percentages) or medians (25th–75th percentile). *p*-values of P‑1 and P‑2 represent the statistical significance of the pairwise comparison of pre-COVID-19 versus COVID-19 year 1 and COVID-19 year 1 versus COVID-19 year 2, respectively

*AS* aortic stenosis, *CABG* coronary artery bypass grafting, *COPD* chronic obstructive pulmonary disease, *HF* heart failure, *ICD* implantable cardioverter defibrillator, *LVEF* left ventricular ejection fraction, *MG* mean gradient, *NYHA* New York Heart Association, *PCI* percutaneous coronary intervention, *TAVI* transcatheter aortic valve implantation, *TIA* transient ischaemic attack

^a^Percentages given of non-missing population: *n* = 47/261, *n* = 56/248, *n* = 42/205 in pre-COVID, year 1 and year 2, respectively

^b^Determined by multidisciplinary heart team assessment

Invasive transaortic mean and peak gradients and LVEDP measurements were available in 98% of patients. Overall median LVEDP was 19 mm Hg (14–25 mm Hg), median transaortic mean gradient (MG) was 43 mm Hg (32–55 mm Hg), and median transaortic peak gradient (PG) was 49 mm Hg (34–65 mm Hg). MG was similar between cohorts, while a tendency for higher PG was observed in the COVID era (PG 46 mm Hg (31–61 mm Hg) vs 51 mm Hg (34–67 mm Hg), *p* = 0.07). LVEDP was significantly higher in the COV-Y1 group compared to PCOV (20 mm Hg (16–26 mm Hg) vs 17 mm Hg (13–24 mm Hg), *p* = 0.01). No difference was found when comparing COV-Y1 and COV-Y2 (Tab. 2). Conversely, pulse pressure was significantly higher in the PCOV group compared to the COV-Y1 group (83 ± 28 mm Hg vs 77 ± 26 mm Hg, *p* = 0.01). Correspondingly, the heart-rate-adjusted ARi was higher in the PCOV group compared to the COV-Y1 cohort.

**Table 2:** Invasive pressures

Invasive pressures	Pre-COVID-19 *n* = 261	COVID-19 year 1 *n* = 241	COVID-19 year 2 *n* = 202	*p*-value (P-1)	*p*-value (P-2)
LVEDP, mm Hg	17 (13–24)	20 (16–26)	19 (14–24)	0.01	0.10
Transaortic mean gradient, mm Hg	41 (31–54)	43 (30–57)	44 (34–55)	0.24	0.85
Transaortic peak gradient, mm Hg	46 (31–61)	51 (34–67)	50 (37–67)	0.07	0.63
Aortic pulse pressure, mm Hg	83 ± 28	77 ± 26	75 ± 27	0.01	0.56
Heart rate adjusted ARi	45 ± 15	42 ± 15	40 ± 15	0.03	0.32

Values are means ± SD or medians (25th–75th percentile). *p*-values of P‑1 and P‑2 represent the statistical significance of the pairwise comparison of pre-COVID-19 versus COVID-19 year 1 and COVID-19 year 1 versus COVID-19 year 2, respectively

*ARi* aortic regurgitation index, *LVEDP* left ventricular end-diastolic pressure

Procedural details are reported in Tab. 3. A total of 28 (4% of the overall cohort) patients underwent a BAV. All procedures were performed with the patient under local anaesthesia. The procedure was performed using transfemoral access in 687 (95%) patients. Transfemoral access was performed less often in the PCOV cohort than in COV-Y1 (92% vs 98%, *p* = 0.01). Admission time, including time in the referring hospital, did not differ between the PCOVand COV-Y1 groups. However, it was significantly lower in COV-Y2 compared to COV-Y1 (median of 4 days (3–7 days) vs 6 days (4–12 days), *p* = 0.01). Admission to the intensive care unit (ICU) was significantly less common in the COV-Y1 group compared to the PCOV group (5% vs 13%, *p* = 0.01).

**Table 3:** Procedural characteristics and clinical outcomes at 30 days

	Pre-COVID-19 *n* = 266	COVID-19 year 1 *n* = 249	COVID-19 year 2 *n* = 205	*p*-value (P-1)	*p*-value (P-2)
*Procedural characteristics*					
BAV	9 (3.4)	12 (4.8)	7 (3.4)	0.41	0.46
TAVI	257 (96.6)	237 (95.2)	198 (96.6)		
Transfemoral access	245 (92.1)	245 (98.4)	197 (96.1)	0.01	0.13
Immediate procedural mortality	2 (0.8)	1 (0.4)	3 (1.5)	0.99	0.29
Emergency cardiac surgery	2 (0.8)	1 (0.4)	2 (1.0)	0.99	0.45
Admission time, days^a^	7 (4–11)	6 (4–12)	4 (3–7)	0.80	0.01
ICU admission	34 (12.8)	13 (5.2)	16 (7.8)	0.01	0.26
*Clinical outcomes at 30 days—VARC‑3*					
All-cause mortality Cardiovascular mortality	6 (2.3) 6 (2.3)	8 (3.2) 7 (2.8)	11 (5.4) 8 (3.9)	0.50 0.78	0.25 0.60
All-cause mortality % of TAVI population^b^	5 (1.9)	6 (2.5)	11 (5.6)	0.66	0.11
Major vascular complication	12 (4.5)	10 (4.0)	8 (3.9)	0.78	0.95
Cardiac structural complication	4 (1.5)	2 (0.8)	4 (2.0)	0.69	0.42
Major bleeding	14 (5.3)	6 (2.4)	10 (4.9)	0.09	0.16
Stroke/TIA	9 (3.4)	9 (3.6)	9 (4.4)	0.89	0.67
Acute kidney injury ≥ II	7 (2.6)	2 (0.8)	5 (2.4)	0.18	0.25
Valve-related surgery or intervention	2 (0.8)	1 (0.4)	2 (1.0)	0.99	0.59
New permanent pacemaker	40 (15.0)	28 (11.2)	19 (9.3)	0.20	0.49
Aortic regurgitation ≥ moderate^c^	19 (7.9)	7 (3.0)	7 (3.7)	0.02	0.07

Values are numbers (percentages) or medians (25th–75th percentile). *p*-values P‑1 and P‑2 represent the statistical significance of the pairwise comparison of pre-COVID-19 versus COVID-19 year 1 and COVID-19 year 1 versus COVID-19 year 2, respectively. Aortic regurgitation was assessed by echocardiogram at discharge. Clinical outcomes are according to Valve Academic Research Consortium (*VARC*)-3 consensus definitions.

*BAV* balloon aortic valvuloplasty, *ICU* intensive care unit, *TAVI* transcatheter aortic valve implantation, *TIA* transient ischaemic attack

^a^Excluding patients with in-hospital mortality

^b^*n* = 5/257, *n* = 6/237, *n* = 11/198 in pre-COVID, year 1 and year 2, respectively

^c^*n* = 19/240, *n* = 7/233, *n* = 7/190 in pre-COVID, year 1 and year 2, respectively

Overall, the 30-day all-cause mortality rate was 4% (*n* = 25) with cardiovascular mortality in 21 of these 25 patients (84%). Thirty-day clinical outcomes per group are described in Tab. 3. No statistically significant differences could be demonstrated for 30-day mortality across the study cohorts. No significant differences were found when comparing other main VARC‑3 endpoints except for AR grade ≥ moderate, which occurred less often in the COV-Y1 group than in the PCOV group (3.0% vs 7.9%, *p* = 0.02). Of note is that in 43% of patients who underwent BAV during the study period, an aortic valve replacement was performed during follow-up.

**Fig. 2:**
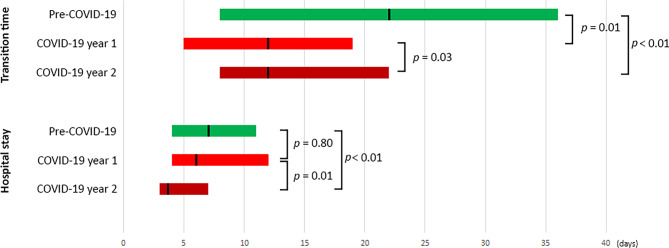
Transition time and length of hospital stay in pre-COVID versus COVID era groups. *Bars* represent interquartile range with left and right margins corresponding to the 25th and 75th percentile, respectively. The *black line* within each bar signifies the group median. Transition time is defined as time between heart team decision and transcatheter aortic valve implantation

Transition times between heart team discussion and procedure and duration of hospital stay are reported in Fig. 2. Median transition time was significantly reduced in COV-Y1 compared with PCOV. In COV-Y2, the length of hospital stay was significantly reduced compared to COV-Y1.

## Discussion

This single-centre experience compared the overall TAVI pathway and clinical and haemodynamic status of patients with symptomatic severe AS or degenerated bioprosthesis undergoing TAVI in three different historical cohorts before and during the COVID-19 pandemic. The main findings are:

1. NYHA functional class did not differ between groups.
2. Haemodynamic status was different, with slightly lower LVEF and higher LVEDP in the COVID era.
3. The need for ICU admission became negligible and overall length of hospital stay shortened.
4. Transition time from heart team discussion to TAVI procedure was reduced over time.
5. Thirty-day clinical outcomes were not significantly different across the different cohorts, except for a lower rate of ≥ moderate AR in the 1st year of COVID.

Age, EuroScore II, NYHA class and kidney function remained similar (Tab. 1) in the pre-COVID and COVID era. This could suggest that the risk profile of patients treated with TAVI/BAV remained stable throughout the study period. However, the overall risk profile was high with frailty present in 47% of the population, and 37% of patients were walking aid dependent. Of note is that in the Netherlands reimbursement for TAVI was available only for high-risk patients at the time of study inclusion. Frailty appeared more frequently in the PCOV period and we noted a lower prevalence of PAD and fewer alternative access TAVI procedures. These findings may hint at a more stringent selection process and referral bias during the pandemic. The COVID-19 pandemic could have resulted in decreased referral rates and/or increased mortality rates in this high-risk population. Indeed, mortality rates in the elderly population increased significantly throughout the COVID era [9]. The number of cases of decompensated AS was higher in the COVID era. Clearly, hospitalisation policies differed throughout the study period and thresholds for heart failure admission may have risen.

The COVID-19 pandemic imposed an unprecedented strain on national healthcare systems and forced re-allocation of resources to cope with the flood of infected patients in general wards and ICUs. In many geographical areas TAVI practice was reduced or even temporarily interrupted [10, 11]. Consequently, cardiovascular societies issued directions on the management of patients referred for structural heart disease interventions [12, 13]. Key recommendations were to treat the most symptomatic AS patients first and to prioritise a streamlined or minimalistic TAVI strategy (without general anaesthesia and limiting ICU care) to limit resources in times of crisis. Our COVID findings are comparable to what was recently reported in the UK. In a large British registry, patients undergoing TAVI in the COVID era had fewer co-morbidities but were more symptomatic and had more depressed LV function [10]. Thirty-day TAVI mortality hazards were similar between groups and length of hospital stay was significantly shorter in the COVID era. Our dataset covered 24 months of the COVID era and may complement this UK registry, which covered only the first 9 months of the pandemic.

We noted differences in quantitative markers of LV function, such as TTE-assessed LVEF and invasively measured LVEDP, that pointed towards more cardiac damage and higher filling pressures in the COVID era (Tab. 1 and 2; [14]). Delaying aortic valve replacement for severe AS may stress the left ventricle and create pressure and eventually also volume overloading. As a result, LV filling pressures rise and EF may decline [2, 15]. Depressed LVEF at baseline is associated with increased risk of mortality after TAVI [16]. Diastolic dysfunction reflected by elevated LVEDP is part of the natural progression of severe AS [14] and is a predictor of mortality and HF [17, 18]. Both decreased LVEF and increased LVEDP may suggest that patients with severe AS in the COVID era were referred in a worse pathophysiological condition. This may stem from a relatively delayed referral pattern in the Netherlands during the COVID-19 pandemic [19].

Compared to the PCOV cohort, transition times from presentation to the heart team until TAVI/BAV procedure and length of hospital stay were significantly shorter in the COVID era, which attests to the efficacy of our optimised and more streamlined TAVI pathway. Streamlined TAVI includes peri-procedural geriatric assessment, local anaesthesia, guidewire-mediated pacing and does not involve systematic post-procedural ICU admission [20]. Furthermore, routine screening of risk factors for conduction disturbances (i.e. electrocardiogram and anatomical markers such as membranous septum length) [21] and optimised implantation techniques (integrated cusp overlap) may have led to earlier discharge. Of note is that the need for ICU admission after TAVI was low and did not impact ICU occupancy, which reached critical thresholds during the 1st year after the COVID outbreak (Tab. 3).

All-cause mortality was similar before and during the COVID pandemic. This was in spite of increased strain on the healthcare system, fewer resources and faster transition times. We observed a higher incidence of AR ≥ moderate in the PCOV population. This is congruent with the invasively measured ARi (which is also higher in the PCOV group). A possible explanation could be the use of different, iterated valves throughout the study period. However, this requires further investigation.

### Limitations

Our study only reports on patients who were referred to our centre for TAVI/BAV. We have no insights into the criteria used by referring cardiologists to refer patients for TAVI. Changing referral patterns may have generated relevant selection bias. Echocardiographic and invasive pressure measurements were site reported and were not evaluated by an independent central core laboratory.

## Conclusion

Patients referred for TAVI during the COVID-19 pandemic more often had slightly depressed LV function and elevated LV filling pressures. The TAVI pathway became more efficient with shorter transition times in the subsequent years. The duration of hospital stay declined over the study period.

### Supplementary Information

Supplementary table 1: Baseline symptom status in the 3 time cohorts. Supplementary table 2: Medical therapy status in the 3 time cohorts
