# Publisher Correction to: Changing haemodynamic status of patients referred for transcatheter aortic valve intervention during the COVID-19 pandemic

**DOI:** 10.1007/s12471-023-01808-w

**Published:** 2023-09-04

**Authors:** Joris F. Ooms, Thijmen W. Hokken, Rik Adrichem, Dilay Gunes, Marjo de Ronde-Tillmans, Isabella Kardys, Jeannette Goudzwaard, Francesco Mattace-Raso, Rutger-Jan Nuis, Joost Daemen, Nicolas M. Van Mieghem

**Affiliations:** 1 Department of Interventional Cardiology, Thoraxcenter, Erasmus University Medical Centre, Rotterdam, The Netherlands; 2 Department of Internal Medicine, Section of Geriatrics, Erasmus University Medical Centre, Rotterdam, The Netherlands

**Correction to:**

**Neth Heart J 2023**

10.1007/s12471-023-01795-y

Figure 2 was inadvertently missing from this article; the figure should have appeared as shown below. The caption to Fig. 2 was inadvertently published below the infographic.

**Fig. 2:**
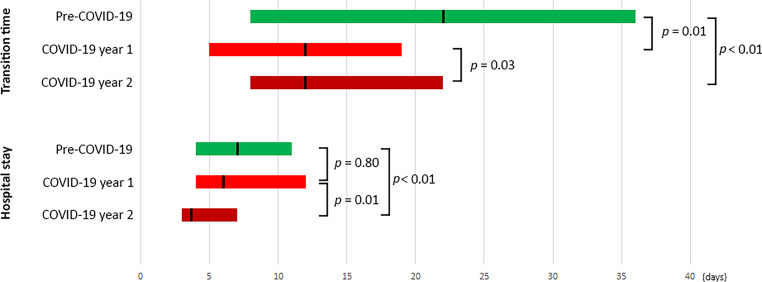
Transition time and length of hospital stay in pre-COVID versus COVID era groups. *Bars* represent interquartile range with left and right margins corresponding to the 25th and 75th percentile, respectively. The *black line* within each bar signifies the group median. Transition time is defined as time between heart team decision and transcatheter aortic valve implantation

The original article has been corrected.

