# Supplementary material for: Changing haemodynamic status of patients referred for transcatheter aortic valve intervention during the COVID-19 pandemic
Source: Neth Heart J. 2023 Jul 27;31(10):399–405. doi: 10.1007/s12471-023-01795-y (PMC10516812; doi:10.1007/s12471-023-01795-y)
Supplement: Supplementary file 1 — Supplementary table 1: Baseline symptom status in the 3 time cohorts. Supplementary table 2: Medical therapy status in the 3 time cohorts [file 12471_2023_1795_MOESM1_ESM.docx]

**Supplementary tables**

| **Table S1. NYHA functional class** | **Pre-COVID-19**  **(n=261)** | **COVID-19 Year 1**  **(n=248)** | **COVID-19 Year 2**  **(n=205)** |
| --- | --- | --- | --- |
| NYHA Class I | 10.0 | 7.3 | 4.4 |
| NYHA Class II | 38.3 | 35.1 | 37.1 |
| NYHA Class III | 33.7 | 35.1 | 38.0 |
| NYHA Class IV | 18.0 | 22.6 | 20.5 |
| Median NYHA Class [25^th^-75^th^ percentile] | 3 [2-3] | 3 [2-3] | 3 [2-3] |

Values are percentages unless mentioned otherwise. NYHA: New York Heart Association

| **Table S2. HF medication** | **Pre-COVID-19**  **(n=266)** | **COVID-19 Year 1**  **(n=249)** | **COVID-19 Year 2 (n=205)** | **p-value**  **(P-1)** | **p-value**  **(P-2)** |
| --- | --- | --- | --- | --- | --- |
| RAAS inhibitors | 55.3 | 52.2 | 51.2 | 0.46 | 0.77 |
| Beta-blocker | 62.8 | 58.6 | 56.6 | 0.28 | 0.66 |
| Mineralocorticoid receptor antagonist | 4.5 | 4.8 | 5.9 | 0.88 | 0.63 |
| Any Diuretic | 59.4 | 59.4 | 56.1 | 0.92 | 0.47 |

Values are percentages unless mentioned otherwise. RAAS: Renin-Angiotensin-Aldosterone System
